# Metabolic Inflammation as a Common Thread in Cardio-Endocrine Diseases: Toward a Unified Therapeutic Framework

**DOI:** 10.7759/cureus.102160

**Published:** 2026-01-23

**Authors:** Nidhi Patil, Neha Uppal, Simranjeet Bedi, Hiral Undhad, Pooja Joshi

**Affiliations:** 1 Emergency Medicine, Lister Hospital, Stevenage, GBR; 2 Internal Medicine, Integrated Medical Care Hospital, Lahore, PAK; 3 Internal Medicine, Kharkiv National Medical University, Kharkiv, UKR; 4 Internal Medicine, Jiangsu University School of Medicine, Zhenjiang, CHN; 5 Internal Medicine, Kanti Devi Medical College Hospital and Research Centre, Mathura, IND

**Keywords:** adipose tissue inflammation, biomarkers, cardio-endocrine diseases, cardiovascular disease, gut microbiota, inflammasome, metabolic inflammation, metaflammation, obesity, type 2 diabetes mellitus

## Abstract

Metabolic inflammation, or metaflammation, has emerged as a unifying mechanism linking cardiovascular and endocrine disorders. This narrative review aimed to synthesize mechanistic, clinical, and therapeutic evidence on how inflammation bridges these domains and to explore prospects for a unified therapeutic framework. We systematically searched PubMed, Scopus, and Web of Science for English-language articles published between 2010 and 2024, yielding 256 relevant articles, of which 115 were included after detailed screening. Studies addressing adipose tissue inflammation, gut microbiota dysbiosis, innate immune activation, mitochondrial dysfunction, clinical biomarkers, and anti-inflammatory therapies were analyzed. Evidence demonstrates that visceral adipose tissue (VAT) inflammation, inflammasome activation, and mitochondrial oxidative stress form shared pathogenic nodes across cardiovascular and endocrine diseases. Clinical correlates, including biomarkers such as IL-6, C-reactive protein (CRP), glycoprotein acetylation (GlycA), and exosomes, provide diagnostic and prognostic insights, while therapeutic convergence has been highlighted by sodium-glucose cotransporter 2 (SGLT2) inhibitors, glucagon-like peptide-1 (GLP-1) receptor agonists, and IL-1β-targeted interventions. Lifestyle modifications such as Mediterranean diets, microbiota-directed therapies, and intermittent fasting further reinforce dual-organ protection. Despite significant advances, limitations remain in translating multi-omics discoveries, biomarker integration, and systems pharmacology into routine practice. Current evidence underscores the need for prospective trials incorporating inflammatory phenotyping, composite cardio-endocrine outcomes, and patient-centered endpoints. Research gaps include the lack of standardized biomarkers, insufficient inclusion of diverse populations, and limited mechanistic insights from physiologically relevant models such as organ-on-chip (OoC) systems and induced pluripotent stem cell (iPSC)-derived organoids. Future studies must prioritize precision-medicine approaches and integrated care models to reduce the global burden of cardio-endocrine disease.

## Introduction and background

Crossroads of metabolism, inflammation, and disease

Metabolic inflammation, or metaflammation, has emerged as a central link between cardiovascular and endocrine disorders. Cardiovascular disease (CVD) remains the leading cause of death worldwide, accounting for approximately 19 million deaths and 438 million disability-adjusted life years (DALYs) in 2021, according to the Global Burden of Disease (GBD) study [1]. Ischemic heart disease (IHD) is the most prevalent contributor, with an estimated 126 million cases and nine million deaths in 2020 [2]. Endocrine disorders substantially contribute to CVD through either direct hormonal imbalances or compensatory mechanisms that alter cardiac and vascular structure and function [3]. In 2019, approximately 1.2 billion people were affected by non-alcoholic fatty liver disease (NAFLD), 463 million by type 2 diabetes mellitus (T2DM), and over 1.3 billion by hypertension (HTN) [4]. Diabetes, in particular, represents a growing global health burden and is among the leading causes of death and disability [4], markedly increasing the risk of myocardial infarction (MI), heart failure, atrial fibrillation, and cardiovascular mortality [5]. This overlap underscores the shared pathophysiological basis of metabolic inflammation and insulin resistance.

**Figure 1:**
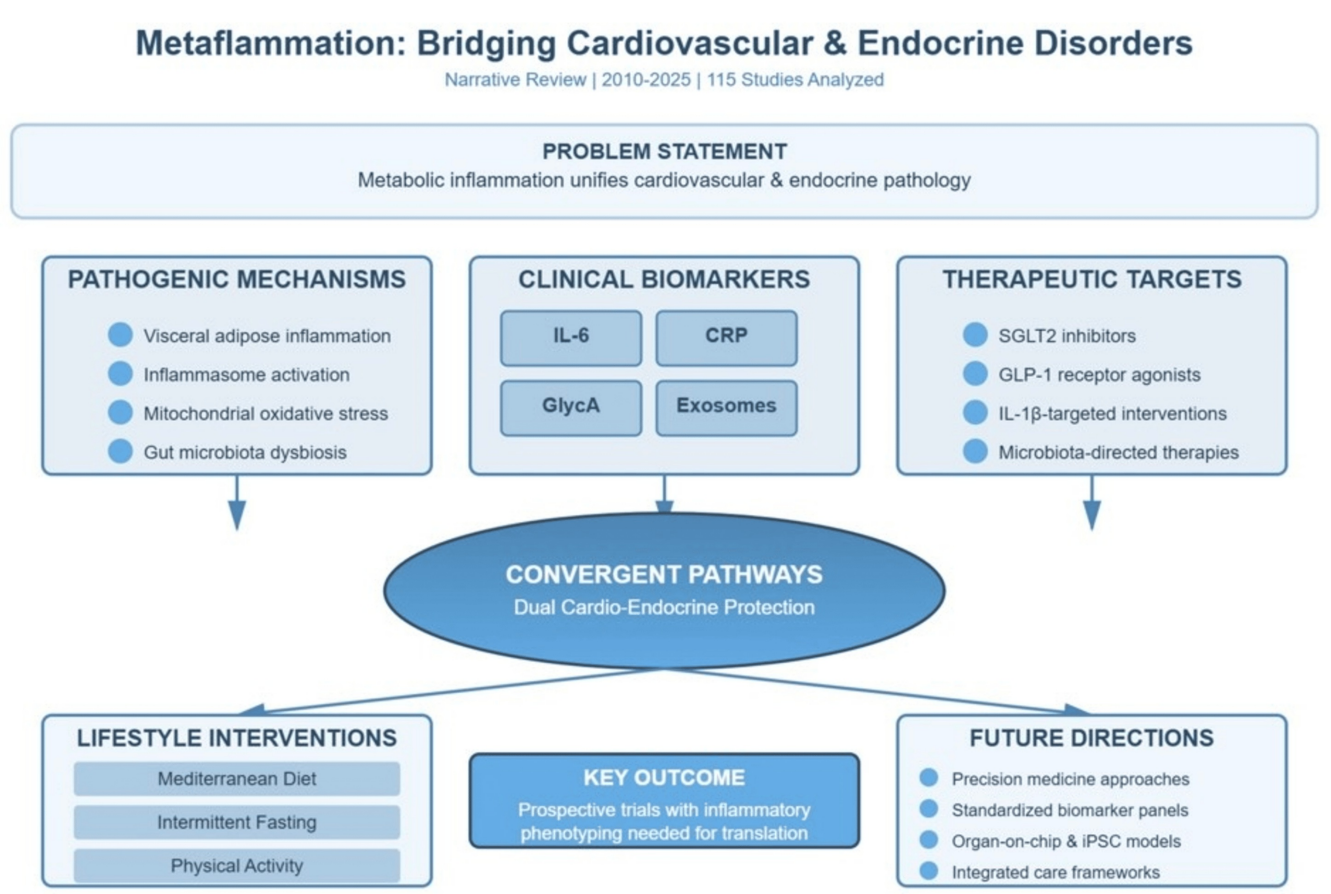
Central illustration of the article summary CRP: C-reactive protein; GLP-1: glucagon-like peptide-1; SGLT2: sodium-glucose cotransporter 2; iPSC: induced pluripotent stem cell; GlycA: glycoprotein acetylation The figure is created by the authors of this study.

The clinical urgency of this mechanistic overlap is further emphasized by evidence linking chronic metabolic inflammation to severe and often fatal cardiovascular outcomes, including acute vascular events and sudden cardiac death, thereby reinforcing the need for an integrated cardio-endocrine framework [1-5]. Figure 1 summarizes the details captured in this review.

Global prevalence of selected cardio-metabolic diseases in 2019 is summarized in Table 1 [4]. This table summarizes the number of individuals affected by NAFLD, T2DM, and HTN, reflecting the large-scale burden of metabolic inflammation-related diseases.

**Table 1:** Global prevalence of selected cardio-metabolic diseases in 2019 NAFLD: nonalcoholic fatty liver disease, T2DM: type 2 diabetes mellitus, HTN: hypertension.

Disease	No. of People Affected Globally (2019) [4]
NAFLD	1.2 billion
T2DM	43.8 million
HTN	18.5 million

The combined impact of cardio-endocrine conditions exerts a major strain on the healthcare system. While the prevalence of diabetes has remained relatively stable, its direct and indirect costs continue to rise [6]. A review by Seuring et al. (2015) highlighted that direct medical expenditures, including drugs and hospitalizations, are substantially higher in high-income countries (HICs) than in low- and middle-income countries (LMICs), ranging from $242 in Mexico to $11,917 in the United States. In LMICs, much of the burden falls on patients through out-of-pocket spending, and data on productivity losses remain limited [5]. For instance, IHD led to 6,700 premature workforce exits in 2015 among individuals aged 45-64 years, equating to an income loss of US $263 million, projected to reach US $426 million by 2030 [7]. In a systematic review of 83 studies, per-episode treatment costs ranged from $500 to $1,500 for CVD and HTN and exceeded $5,000 for coronary heart disease and stroke [8].

Defining metabolic inflammation

Inflammation plays a pivotal role in the pathogenesis of metabolic syndrome (MetS) and cardio-endocrine diseases. Adipose tissue macrophages (ATMs) are central regulators of adipose tissue homeostasis and are the dominant drivers of inflammation in obesity [9]. Normally, macrophages constitute approximately 10% of adipose tissue cells, but this proportion can increase to nearly 50% in obesity [10]. Lipotoxicity leads to the release of toxic lipid species, including saturated free fatty acids, ceramides, and oxidized lipids, into adipose tissue and circulation, which in turn reprogram macrophages toward a pro-inflammatory phenotype [11,12].

Macrophages respond to both microbial and stress-related signals through pattern recognition receptors (PRRs), which detect pathogen-associated molecular patterns (PAMPs) and damage-associated molecular patterns (DAMPs) [13]. A key PRR pathway implicated in obesity-induced inflammation is the nucleotide-binding oligomerization domain, leucine-rich repeat, and pyrin domain-containing protein 3 (NLRP3) inflammasome, which activates caspases and triggers the release of IL-1β and IL-18, perpetuating chronic inflammation [14]. Endoplasmic reticulum (ER) stress links lipotoxicity to inflammation by activating the unfolded protein response (UPR). If unresolved, ER stress exacerbates cytokine production, insulin resistance, and cell death [15].

Although substantial research has clarified these mechanisms, many molecular drivers of metabolic diseases remain incompletely defined. Advances in genetics, epigenomics, transcriptomics, proteomics, and metabolomics now allow systems-level investigation of diseases, providing comprehensive insights into the multi-organ pathways underlying these conditions [16].

Rationale for a unified framework

Historically, cardiovascular and endocrine conditions have been studied in isolation, yet accumulating evidence underscores their interconnectedness through shared metabolic-inflammatory mechanisms [17]. Treating these diseases separately risks overlooking the synergistic effects of comorbidities such as obesity, diabetes, and CVD, which exacerbate each other and amplify the global health burden [18].

A systems biology perspective offers a framework to integrate genetic, molecular, and organ-level interactions, highlighting how insulin resistance, lipid dysregulation, immune activation, and cross-organ signaling form pathogenic networks driving both endocrine and CVDs [16]. This holistic view is essential for the development of diagnostics and therapies that simultaneously address both domains.

This unified approach was supported by the concept of therapeutic convergence. Several therapies demonstrate dual benefits: sodium-glucose cotransporter 2 (SGLT2) inhibitors, initially developed for glycemic control, also reduce heart failure hospitalizations and protect renal function [18]; IL-1β blockers such as canakinumab and NLRP3 inflammasome inhibitors show promise across metabolic and cardiovascular outcomes [19,20]; and long-term lifestyle interventions combining calorie restriction, weight loss, and structured exercise improve cardiovascular risk factors in individuals with T2DM [21].

Cardio-endocrine diseases share overlapping mechanisms with metabolic inflammation as a unifying driver. The growing prevalence and economic costs demand an integrated perspective that bridges cardiology and endocrinology. Therefore, this review explores the mechanistic links between metabolic inflammation and cardio-endocrine disease, clinical correlates and biomarkers, and opportunities for therapeutic convergence.

Mechanistic pathways linking metabolic inflammation to cardio-endocrine diseases

Adipose Tissue Inflammation and Adipokines

Obesity represents a chronic inflammatory state that drives cardiometabolic dysfunction through adipose tissue derangements, immune imbalance, and vascular injury. Adipose tissue is now recognized as an active endocrine organ that produces adipokines that regulate immunity, metabolism, and vascular tone [22]. In lean states, ATMs are predominantly anti-inflammatory M2 cells that support insulin sensitivity via IL-10 and adiponectin [23,24]. In obesity, adipocyte hypertrophy, hypoxia, and necrosis recruit pro-inflammatory M1 macrophages, increasing tumor necrosis factor-alpha (TNF-α) and IL-6, which impair insulin receptor signaling in the liver and muscle [23,25].

When elevated, adipokines such as leptin and resistin promote vascular dysfunction; leptin drives vascular smooth muscle proliferation and atherogenesis, while resistin enhances oxidative stress and endothelial injury [26,27]. Meanwhile, adiponectin levels decrease, diminishing vascular protection. Depot-specific activity further amplifies inflammation, as visceral adipose tissue (VAT) is more metabolically active and pro-inflammatory than subcutaneous adipose tissue (SAT) [28]. VAT-derived cytokines disrupt hepatic metabolism via portal circulation, worsening dyslipidemia. Furthermore, amplifiers such as mitochondrial DNA release sustained inflammation, while gut microbiota-derived metabolites like trimethylamine N-oxide (TMAO) contribute independently to systemic inflammation and cardiovascular risk [29]. Clinical studies confirm VAT’s pathogenic role of VAT; for example, omentectomy added to Roux-en-Y gastric bypass did not improve insulin sensitivity but reduced C-reactive protein (CRP) levels after 12 months [30].

Gut Microbiota and Metabolic Endotoxemia

The gut microbiota profoundly influences metabolic and cardiovascular health. Obese individuals typically exhibit reduced microbial diversity, higher abundance of pro-inflammatory gram-negative bacteria, and reduced short-chain fatty acid (SCFA)-producing microbes [31]. Increased intestinal permeability enables lipopolysaccharide (LPS) translocation into the circulation, triggering Toll-like receptor 4 (TLR4) signaling in adipose tissue, liver, and vasculature, promoting insulin resistance and systemic inflammation [32,33].

Altered microbial metabolism enhances TMAO generation, which is strongly linked to atherosclerosis and adverse cardiovascular outcomes [34]. Modulation strategies, such as probiotics, prebiotics, and dietary fiber, have demonstrated limited enhancements in insulin sensitivity and inflammatory markers [35]. Simultaneously, pancreatic islets experience low-grade inflammation in obesity, characterized by increased IL-1β and TNF-α levels, which hinder insulin secretion [36]. Hyperglycemia-induced glucotoxicity worsens cardiomyocyte function [37]. Mendelian randomization analyses support a potential causal role for TMAO in CVD pathogenesis [38], though its utility as a therapeutic target remains under investigation.

Innate Immune Activation in the Heart and Pancreas

The innate immune system senses metabolic danger signals via inflammasomes. The NLRP3 inflammasome activates caspase-1, driving the release of IL-1β and IL-18 [39]. In myocardial tissue, NLRP3 activity promotes cardiomyocyte hypertrophy, apoptosis, and fibrosis, thereby contributing to adverse remodeling [40]. Patients with high IL-1β levels post-MI demonstrate worse ventricular function over one year [41].

Obesity-related triggers of NLRP3 activation include saturated fatty acids, cholesterol crystals, and mitochondrial deoxyadenosine monophosphate (DAMPs) [42]. In the pancreas, inflammasome activation induces β-cell apoptosis and reduces insulin secretion [43]. Preclinical studies have revealed that NLRP3 inhibition improves glycemic control and attenuates cardiac remodeling [44]. Imaging studies have demonstrated that epicardial adipose tissue inflammasome activity correlates with diastolic dysfunction independent of BMI. Notably, circulating LPS levels rise two- to threefold in obesity, potentiating inflammasome activation [45].

Mitochondrial Dysfunction and Oxidative Stress

Mitochondrial dysfunction represents a unifying mechanism in both endothelial and β-cell injury. Obesity elevates reactive oxygen species (ROS) levels, impairing nitric oxide bioavailability and endothelial vasodilation [46,47]. ROS damages mitochondrial DNA (mtDNA), reduces ATP production, and triggers β-cell apoptosis [48]. Released mtDNA acts as a DAMP, activating PRRs, such as TLR9, further stimulating NLRP3 inflammasome signaling [49].

The consequences include diastolic dysfunction of the heart and impaired pancreatic insulin secretion. Experimental therapies targeting mitochondrial biogenesis and antioxidant pathways show promise in mitigating these effects [47-50].

**Figure 2:**
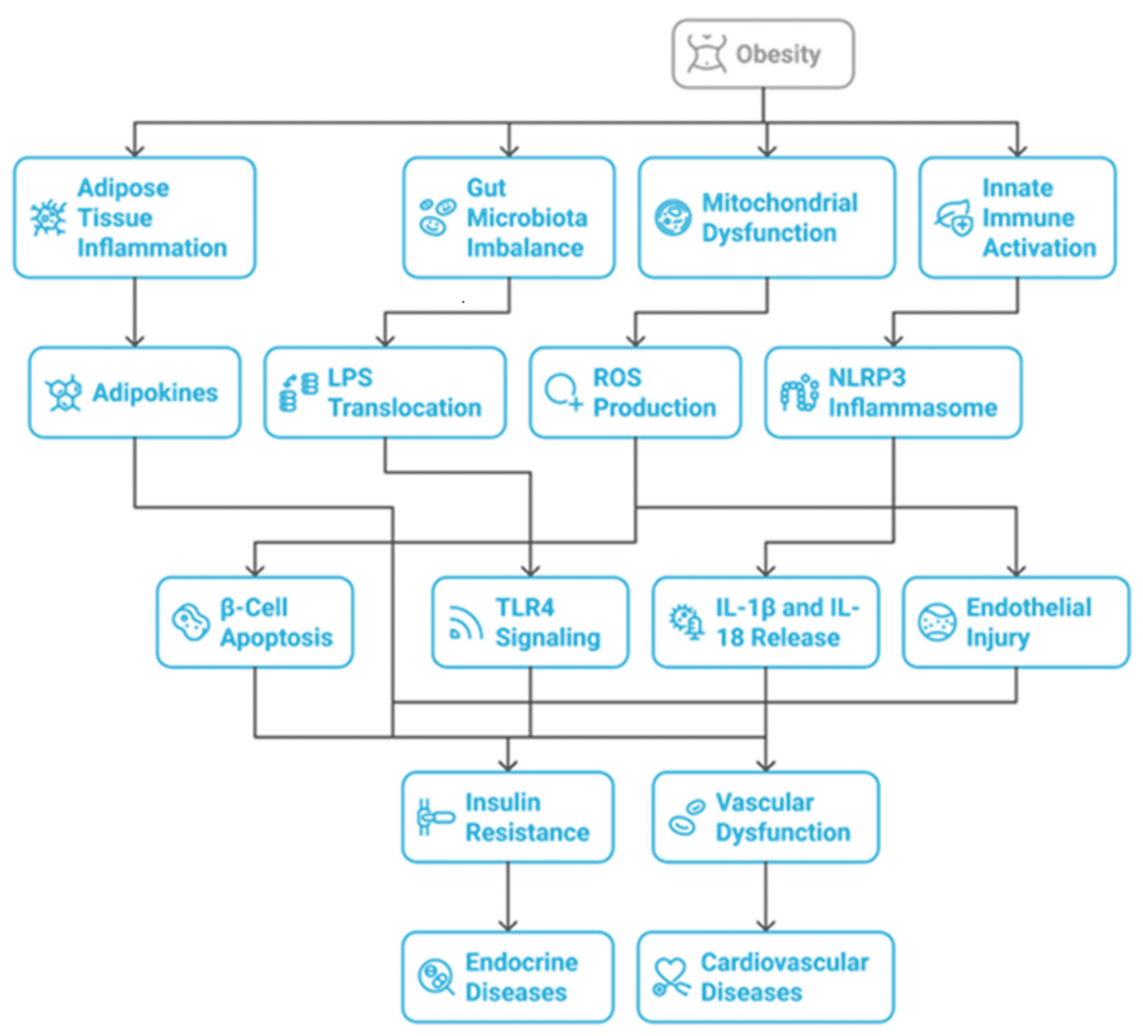
Metabolic inflammation and cardio-endocrine disease ROS: reactive oxygen species; LPS: lipopolysaccharide The figure is created by the authors of this study.

Figure 2 illustrates how obesity-induced inflammation alters adipose tissue, gut microbiota, innate immunity, and mitochondrial function. Together, these processes generate systemic metabolic stress that drives both cardiovascular (atherosclerosis, cardiac remodeling, endothelial dysfunction) and endocrine (insulin resistance, β-cell failure) complications.

## Review

Clinical correlates and biomarkers: evidence from human studies

Inflammatory biomarkers are measurable biological signals, including circulating molecules, tissue-derived factors, and imaging features that capture chronic low-grade inflammation driven by metabolic dysfunction. Their clinical value lies in their diagnosis, risk stratification, and disease monitoring across cardiovascular and endocrine disorders. As emphasized by Menzel et al. (2021), quantifiable biomarkers provide standardized and reproducible measures with defined reference ranges, allowing clinicians to stratify risk, monitor progression, and evaluate therapeutic response [51]. For instance, a high level of high-sensitivity CRP (hsCRP) (>3 mg/L) shows that a person is at a higher risk of heart disease, while measuring IL-6 or glycoprotein acetylation (GlycA) over time shows how much inflammation is going on in the body. Quantifiable inflammatory biomarkers are broadly classified into early response cytokines (IL-6, IL-1β, IL-8, IFN-γ, and TNF-α) and acute-phase proteins (APPs), such as CRP, serum amyloid A (SAA), and haptoglobin [51]. CRP remains the most clinically validated marker; levels may rise 10-100-fold within six to 72 hours of tissue injury, with values >100 μg/mL indicating severe inflammation and poor prognosis [52]. However, specificity increases when CRP is interpreted alongside complementary markers, such as IL-6, GlycA, or adipokines [53,54]. The Multi-Ethnic Study of Atherosclerosis (MESA) demonstrated that IL-6 was a stronger predictor of incident HTN than CRP [53], whereas GlycA provided superior specificity for metabolic inflammation compared to CRP [54]. Collectively, these findings underscore the practicality of CRP while highlighting the mechanistic insights offered by emerging biomarkers.

Novel indices, such as the CRP and triglyceride glucose index, have recently been explored as composite markers of inflammation and insulin resistance, showing predictive value for adverse outcomes in diverse populations [55]. hsCRP further enhances prognostic accuracy, predicting both initial cardiovascular events in healthy individuals and recurrent events in those with coronary syndromes [56]. Advances in multiplex technologies now permit the simultaneous assessment of multiple biomarkers, expanding clinical application [57].

Genomics: Genome-wide studies have found connections between inflammation and diseases of the heart and endocrine system. A meta-analysis by the Genetics Consortium (2012) showed that the IL-6 receptor aspartate 358 alanine variant (IL6R Asp358Ala) reduces systemic inflammation and coronary heart disease risk, directly implicating IL-6 signaling as a therapeutic target [58].

Proteomics: Large-scale proteomic profiling has revealed inflammatory protein networks that are predictive of cardiovascular outcomes. In the Framingham Heart Study, proteins such as growth differentiation factor (GDF-15), IL-6 pathway mediators, and TNF-receptor superfamily members were independently associated with mortality [59].

Metabolomics/lipidomics: These platforms capture dynamic biochemical alterations, including lipid and amino acid profiles, that drive inflammatory processes. Lipidomics supports biomarker discovery by identifying lipid-based mediators of insulin resistance, obesity, and CVD [60].

Exosomes: Small extracellular vesicles with bioactive molecules (proteins, lipids, and miRNAs) regulate inflammatory and metabolic pathways. Exosomes play a role in both protective processes (such as cardioprotection and endothelial proliferation) and harmful outcomes (like vascular inflammation and myocardial injury), making them good candidates for biomarkers and therapeutic vectors [61].

Molecular imaging techniques increasingly allow the in vivo visualization of systemic inflammation. Fluorodeoxyglucose positron emission tomography/computed tomography (FDG-PET/CT) has been validated for diagnosing and monitoring inflammatory conditions [62]. In post-acute myocardial infarction (AMI) patients, CT-based profiling demonstrated increased attenuation of perivascular (PVAT) and epicardial adipose tissue (EAT), indicating a shift towards a pro-inflammatory, edematous phenotype compared with stable coronary artery disease (CAD), which highlights the role of imaging in assessing residual vascular inflammation [63]. Similarly, 18F-FDG PET/CT studies have shown that VAT uptake correlates with CAD severity and carotid inflammation [64]. Advanced MR-based methods, including magnetic resonance imaging (MRI), magnetic resonance spectroscopy (MRS), and magnetic resonance elastography (MRE), provide robust, noninvasive tools for quantifying hepatic and muscular lipids, mitochondrial oxidative metabolism, and fibrogenesis in NAFLD. These modalities correlate well with histology, providing helpful details about systemic lipotoxicity and inflammation [65,66].

Table 2 summarizes the overview of studies assessing IL-6, CRP, and other inflammatory markers in diabetes and cardiovascular risk. This table summarizes major human studies evaluating inflammatory biomarkers and their predictive value for cardio-endocrine diseases.

**Table 2:** Overview of studies assessing IL-6, CRP, and other inflammatory markers in diabetes and cardiovascular risk ADVANCE RCT: Action in Diabetes and Vascular Disease: Preterax and Diamicron Modified-Release Controlled Evaluation, randomized controlled trial; T2DM: type 2 diabetes mellitus; CVD: cardiovascular disease; IL-6: interleukin-6; CRP: C-reactive protein; LPS: lipopolysaccharide; hs-CRP: high-sensitivity CRP; MI: myocardial infarction; MetS: metabolic syndrome; IL-6R: interleukin-6 receptor; ICAM-1: intercellular adhesion molecule-1; CARDIA: Coronary Artery Risk Development in Young Adults Study; E-selectin: endothelial selectin; MR: Mendelian randomization; FU: follow-up

Ref	Study / First Author (Year)	Design & Population	Markers Studied	Key Findings
[67]	ADVANCE RCT – Lowe G et al. (2014)	RCT; T2DM with CVD/risk factors	IL-6, CRP, fibrinogen	IL-6 improved prediction of macrovascular events & mortality; CRP & fibrinogen did not.
[68]	HUNT2 – Løfblad L et al. (2021)	Prospective cohort: general population with/without diabetes	IL-6, CRP	Both associated with CV mortality; only CRP remained independently predictive.
[69]	LPS Human Inflammation Model – Mehta NN et al. (2012)	RCT; healthy participants	Systemic inflammation markers	LPS induced insulin resistance, adipose inflammation, and lipid disturbances resembling obesity/T2D/CVD.
[70]	Sharif S et al. (2021)	Prospective cohort: high-risk T2DM, median FU 7.8 yrs	hs-CRP	Elevated hs-CRP predicted vascular & all-cause mortality, but not MI or stroke.
[71]	Framingham Heart Study – Dallmeier D et al. (2012)	Cross-sectional; community cohort (984 with MetS)	Multiple biomarkers	MetS significantly associated with most biomarkers except osteoprotegerin.
[72]	UK Biobank MR Study: Georgakis MK et al. (2022)	Mendelian randomization; large cohort	IL-6R signaling, hs-CRP	Genetically predicted IL-6R signaling modestly ↑ CVD risk, independent of baseline hsCRP.
[73]	CARDIA Study	Prospective cohort: young adults	ICAM-1, E-selectin	Higher levels predicted incident T2D; improved risk prediction beyond clinical scores.

Therapeutic convergence: targeting metabolic inflammation for dual organ protection

Pharmacological targeting of metabolic inflammation is emerging as a complementary strategy to traditional lipid-lowering therapies, addressing the “residual inflammatory risk” that persists in many patients with cardiometabolic disease [74]. The therapeutic goal is not restricted to isolated pathways but rather involves modulating the chronic inflammatory milieu that drives both cardiovascular and endocrine disorders. Interventions include cytokine-neutralizing monoclonal antibodies (e.g., IL-1β inhibitors such as canakinumab), broad-spectrum anti-inflammatories (e.g., colchicine), and metabolic agents with secondary anti-inflammatory actions (e.g., SGLT2 inhibitors and glucagon-like peptide-1 (GLP-1) receptor agonists) [75-77].

The CANTOS trial (Ridker et al., 2019) provided the first proof-of-concept that selective anti-inflammatory therapy reduces cardiovascular events independently of lipid-lowering. In patients with prior MI and elevated hsCRP (≥2 mg/L), canakinumab (150 mg every three months) reduced hsCRP levels by 37% and lowered major cardiovascular event rates by 15% (HR 0.85, P=0.021) without affecting lipid levels [75]. The trial validated inflammation as a therapeutic target, akin to cholesterol, notwithstanding its restricted clinical applicability owing to expense and heightened infection risk.

Subsequent trials have explored broader, cost-effective agents. The COLCOT trial (Tardif et al., 2019) showed that low-dose colchicine (0.5 mg/day) reduced cardiovascular events after MI (5.5% vs. 7.1%; HR 0.77, P=0.02), mainly through reductions in stroke and revascularization [76]. The LoDoCo-MI analysis further demonstrated the potential of colchicine in lowering residual inflammatory risk, with a 64% higher likelihood of achieving hsCRP ≤1 mg/L, consistent with reduced cardiovascular risk [77].

Together, these trials establish that targeting inflammation, particularly IL-1β, and downstream pathways, such as IL-6 and TNF-α, represents a promising adjunct to lipid management in cardiovascular prevention [78]. Table 3 details the anti-inflammatory trials targeting residual inflammatory risk post-MI.

**Table 3:** Anti-inflammatory trials targeting residual inflammatory risk post-MI CANTOS: Canakinumab Anti-inflammatory Thrombosis Outcomes Study; MI: myocardial infarction; hsCRP: high-sensitivity CRP; SC: subcutaneous; q3m: every three months; CV: cardiovascular; MACE: major adverse cardiovascular events; HR: hazard ratio; COLCOT: Colchicine Cardiovascular Outcomes Trial; LoDoCo-MI: Low-Dose Colchicine Post-MI Study; OR: odds ratio

Trial	Population	Intervention	Comparator	Primary Endpoint	Key Findings	Limitations
CANTOS (Ridker et al., 2019) [75]	10,061 patients with prior MI and hsCRP ≥2 mg/L	Canakinumab (50, 150, 300 mg SC q3m)	Placebo	Composite of nonfatal MI, stroke, CV death	150 mg: 15% MACE reduction (HR 0.85; P=0.021); ↓ hsCRP 37%; no lipid changes	High cost; parenteral; ↑ infection risk
COLCOT (Tardif et al., 2019) [76]	4,745 patients ≤30 days post-MI	Colchicine 0.5 mg/day	Placebo	Composite of CV death, arrest, MI, stroke, urgent angina	5.5% vs 7.1% (HR 0.77; P=0.02); benefit via ↓ stroke/revascularization	Short follow-up; early post-MI focus
LoDoCo-MI (2022 pooled analysis) [79]	429 post-MI patients with hsCRP data	Colchicine 0.5 mg/day	Placebo	hsCRP biomarker	OR 1.64 for hsCRP ≤1 mg/L (P=0.024)	Biomarker-driven outcomes

Beyond glucose control, metabolic agents such as SGLT2 inhibitors, GLP-1 receptor agonists, and thiazolidinediones (TZDs) exert significant anti-inflammatory and cardiovascular benefits. SGLT2 inhibitors reduce oxidative stress and inflammation by attenuating mitochondrial dysfunction, downregulating NADPH oxidase, and lowering TNF-α and IL-6 levels. These mechanisms enhance endothelial function and improve outcomes in both heart failure with preserved ejection fraction (HFpEF) and heart failure with reduced ejection fraction (HFrEF) [80,81]. TZDs (pioglitazone and rosiglitazone) activate PPARγ, inhibit NF-κB signaling, and lower TNF-α and CRP levels, enhancing insulin sensitivity and vascular function; however, their application is constrained by fluid retention [82,83]. Table 4 details the major cardiovascular outcome trials of GLP-1 receptor agonists.

**Table 4:** Major cardiovascular outcome trials of GLP-1 receptor agonists LEADER: Liraglutide Effect and Action in Diabetes: Evaluation of Cardiovascular Outcome Results; T2DM: type 2 diabetes mellitus; CV: cardiovascular; HR: hazard ratio; CI: confidence interval; MACE: major adverse cardiovascular events; REWIND: Researching Cardiovascular Events With a Weekly Incretin in Diabetes; GLP-1: glucagon-like peptide-1

Trial	Population	Intervention	Follow-up	Key Results
LEADER (Marso et al., 2016) [84]	9,340 T2DM, high CV risk	Liraglutide 1.8 mg/day	3.8 yrs	HR 0.87 (95% CI, 0.78–0.97); 13% ↓ MACE; 22% ↓ CV death
REWIND (Gerstein et al., 2019) [85]	9,901 T2DM (majority primary prevention)	Dulaglutide 1.5 mg weekly	5.4 yrs	HR 0.88 (95% CI, 0.79–0.99); 12% ↓ MACE

Lifestyle and dietary interventions offer complementary anti-inflammatory benefits. Mediterranean and plant-based diets diminish CRP and IL-6 levels, enhance endothelial function, and decrease cardiovascular events [86-88]. High-fiber diets decrease IL-6 and TNF-α-R2 levels, supporting cytokine-targeted anti-inflammatory effects [89]. Modulating microbiota via probiotics and prebiotics affects the gut-heart-pancreas axis, with meta-analyses indicating enhancements in glycemic control and inflammation in type 2 diabetes [90,91]. Intermittent fasting activates a "metabolic switch" that transitions the body to ketone metabolism. This process lowers systemic inflammation by lowering NLRP3 inflammasome activity and oxidative stress [92-94].

Traditional single-target therapies inadequately address the complex, multi-node networks driving cardiometabolic diseases. Systems pharmacology integrates network biology and pharmacology to elucidate the impact of drugs on interconnected inflammatory and metabolic pathways. This opens up new ways to use drugs [95,96]. Polypharmacology strategies exploit the synergistic targeting of hubs, such as the NLRP3 inflammasome and GLP-1 signaling, offering dual benefits in vascular and metabolic injury [96]. This network-based model has potential for future therapeutic interventions. Table 5 details the key specialized terms used in this review.

**Table 5:** Glossary of key specialized terms used in this review TLR4: Toll-like receptor 4; CVD: cardiovascular disease; SGT2: sodium-glucose cotransporter 2; GLP-1 RAs: glucagon-like peptide-1 receptor agonists; LPS: lipopolysaccharide

Term	Brief Definition	Key References
Metaflammation	Chronic, low-grade systemic inflammation driven by metabolic dysfunction that underlies cardio-endocrine disease.	[9,14]
DAMPs (Damage-Associated Molecular Patterns)	Endogenous molecules released from stressed or injured cells that activate sterile inflammatory pathways.	[13,49]
PAMPs (Pathogen-Associated Molecular Patterns)	Microbial-derived signals that trigger innate immune responses via pattern-recognition receptors.	[13]
PRRs (Pattern Recognition Receptors)	Innate immune receptors (e.g., TLRs, NLRs) that detect PAMPs and DAMPs to initiate inflammation.	[13]
Inflammasome	Multiprotein complex (e.g., NLRP3) that activates caspase-1 and promotes IL-1β/IL-18–mediated inflammation.	[14,39]
Metabolic Endotoxemia	Chronic elevation of circulating LPS due to increased gut permeability, promoting insulin resistance and CVD risk.	[32,33]
Adipose Tissue Macrophages (ATMs)	Immune cells in adipose tissue that shift to a pro-inflammatory phenotype in obesity, driving metaflammation.	[9,23]
Residual Inflammatory Risk	Persistent inflammatory burden despite optimal lipid control, contributing to ongoing cardiometabolic risk.	[74,75]
Therapeutic Convergence	Use of interventions (e.g., SGLT2 inhibitors, GLP-1 RAs) that simultaneously benefit cardiovascular and endocrine systems by targeting shared pathways.	[80,84]

Future directions and a unified framework for research and practice

Advances in high-yield research such as biomarkers, proteomics, molecular imaging, and therapeutic convergence have significantly expanded our understanding of inter-organ crosstalk in cardiometabolic syndromes, providing a basis for novel translational approaches that move beyond traditional reductionist models. Emerging bioengineered platforms such as organ-on-chip (OoC) systems, which replicate the structural and functional integrity of human tissues through bio-inspired microfluidic devices, enable continuous perfusion, controlled shear stress, and mechanical stimulation, thereby offering physiologically relevant insights into CVD mechanisms that traditional animal studies fail to capture [18,97-99]. In parallel, induced pluripotent stem cell (iPSC)-derived organoids provide patient-specific, three-dimensional models of inflammation and metabolic dysfunction, capable of recapitulating chronic disease processes and inter-organ interactions with higher fidelity than two-dimensional cultures, although challenges such as vascularization limits and central necrosis persist [100-102].

Beyond experimental models, advances in prognostic tools-particularly the Integrated Prognostic Scoring (IPS) system, which incorporates metabolic and inflammatory markers such as lactate dehydrogenase (LDH), alkaline phosphatase (ALP), lymphocyte count, monocyte-to-lymphocyte ratio (MLR), systemic inflammation response index (SIRI), neutrophil-to-lymphocyte ratio (NLR), and red cell distribution width (RDW), have demonstrated superior risk prediction for cardiovascular and all-cause mortality compared with individual biomarkers. These findings underscore the need for standardized, scalable assays that unify cardiovascular and endocrine risk prediction [103,104]. These tools reflect a shift toward precision medicine by integrating systemic inflammation into outcome stratification, a shift reinforced by evidence that CVD is increasingly recognized not merely as a primary cardiac disorder but also as a systemic syndrome involving inter-organ signaling, chronic inflammation, and multi-level metabolic dysfunction across adipose tissue, liver, kidney, and skeletal muscle [105,106].

Translational gaps remain, however, as conventional clinical trial endpoints often fail to capture the systemic nature of cardiometabolic disease, emphasizing the need to rethink trial design by incorporating inflammatory biomarkers, organ crosstalk metrics, and patient-centered outcomes, such as quality of life. A more integrative approach would also involve the development of composite outcomes to reflect “cardio-endocrine syndrome,” capturing the joint contributions of obesity, dyslipidemia, HTN, and diabetes, and their interaction with neurohormonal and inflammatory signaling [107,108]. Recruitment of at-risk populations with systemic inflammation phenotypes is particularly critical, as demonstrated in a National Health and Nutrition Examination Survey (NHANES)-based cohort study, where adults with undiagnosed cardiometabolic disease showed high levels of systemic inflammation, reflecting a missed opportunity for cardiovascular prevention [109].

Clinical correlates and therapeutic convergence: an integrated perspective

Biomarker Classes and Clinical Utility

Clinical correlates of metabolic inflammation provide a critical translational bridge between mechanistic pathways and bedside application. Circulating biomarkers such as IL-6, CRP, GlycA, and extracellular vesicles (exosomes) offer robust diagnostic and prognostic value across cardio-endocrine diseases. IL-6 reflects upstream inflammatory signaling, while CRP serves as a downstream acute-phase reactant and remains the most widely validated marker in cardiovascular risk stratification [51,56]. GlycA, which captures cumulative GlycA, provides a stable index of chronic systemic inflammation and demonstrates superior specificity for metabolic inflammation compared with CRP alone [54].

Exosomes extend biomarker utility by enabling real-time assessment of inter-organ communication, carrying microRNAs, lipids, and proteins that reflect myocardial stress, endothelial dysfunction, and β-cell injury [61]. Together, these biomarkers support risk stratification, inflammatory phenotyping, and therapeutic monitoring, particularly in individuals with overlapping cardiometabolic conditions [53,59].

Molecular Imaging of Inflammation

Advances in molecular imaging increasingly allow the in vivo visualization of inflammatory burdens. Modalities such as 18F-FDG PET/CT and advanced magnetic resonance techniques enable detection of metabolically active adipose depots, vascular inflammation, and myocardial remodeling [62-66]. Imaging-based inflammatory phenotyping complements circulating biomarkers by localizing disease activity, thereby improving precision in risk prediction and therapeutic targeting. For example, increased uptake in visceral and EAT has been associated with CAD severity and residual inflammatory risk after MI [63,64].

Therapeutic Convergence in Cardio-Endocrine Disease

The convergence of cardiovascular and endocrine therapeutics reflects growing recognition of shared inflammatory mechanisms. SGLT2 inhibitors and GLP-1 receptor agonists, initially developed for glycemic control, have demonstrated consistent cardiovascular protection, mediated in part through reductions in oxidative stress, inflammasome activation, and endothelial dysfunction [80,81,84,85].

In parallel, IL-1β-targeted interventions, supported by evidence from the CANTOS trial, provide proof-of-concept that inflammation itself is a modifiable therapeutic target, independent of lipid lowering [75]. Downstream suppression of IL-6 and TNF-α signaling further reinforces the relevance of inflammasome biology in both cardiovascular and metabolic disease progression [78].

Lifestyle interventions strengthen this paradigm of therapeutic convergence. Mediterranean dietary patterns, microbiota-directed therapies, and intermittent fasting have been shown to attenuate systemic inflammation, reduce CRP and IL-6 levels, and improve cardiometabolic outcomes [86-94]. These results highlight the necessity of incorporating both pharmacological and non-pharmacological approaches within a cohesive cardio-endocrine framework.

Translational Challenges and Future Directions

Despite significant advances, important limitations remain in translating multi-omics discoveries, biomarker integration, and systems pharmacology into routine clinical practice. Heterogeneity in biomarker thresholds, lack of standardized inflammatory phenotyping, and insufficient validation across diverse populations continue to limit generalizability [103-105,109].

Current evidence points out the importance of prospective trials incorporating inflammatory phenotyping, composite cardio-endocrine outcomes, and patient-centered endpoints that capture quality of life alongside hard clinical events [107,108]. This approach is necessary to transcend single-organ paradigms and develop a cohesive cardio-endocrine model that can effectively address the intricate, interrelated nature of metabolic inflammation and its systemic ramifications [105,106].

Genomic insights further reinforce the “common soil” hypothesis by identifying overlapping loci that underpin insulin resistance, atherosclerosis, and vascular dysfunction, with genome-wide association studies (GWAS) identifying approximately 600 loci linked to type 2 diabetes, more than 200 linked to CVD, and overlapping regions such as IRS1 that directly implicate insulin resistance pathways in cardiometabolic comorbidity [110-112]. Therefore, future research should emphasize phenotype-directed management strategies, as recent work by Bianchi et al. (2025) demonstrated distinct inflammatory phenotypes in cardiometabolic disease, with varying levels of IL-6, IL-8, and TNF-α corresponding to different burdens of CVD and comorbidity, which points to the importance of stratified anti-inflammatory therapies [113]. Translating these insights into practice requires not only novel biomarkers and genetic tools but also restructured care delivery models. Integrated care frameworks, such as the CINEMA (Center for Integrated and Novel Approaches in Vascular-Metabolic Disease) program, have already shown benefits by combining guideline-based therapies with multidisciplinary coordination to achieve improvements in BMI, blood pressure, HbA1c, cholesterol, and predicted 10-year cardiovascular risk [114]. Similarly, cluster randomized controlled trials have highlighted the importance of coordinated care teams in enhancing adherence to high-intensity statins, RAAS inhibitors, SGLT2 inhibitors, and GLP-1 receptor agonists, with improvements in morbidity and mortality outcomes despite challenges in patient compliance [115]. Table 6 summarizes therapeutic convergence in cardiometabolic diseases.

**Table 6:** Therapeutic convergence in cardiometabolic disease CVD: cardiovascular disease; RCT: randomized controlled trial; MACE: major adverse cardiovascular events; T2DM: type 2 diabetes mellitus; EMPA-REG OUTCOME: Empagliflozin Cardiovascular Outcome Event Trial in Type 2 Diabetes Mellitus Patients; CANVAS: Canagliflozin Cardiovascular Assessment Study; DECLARE–TIMI 58: Dapagliflozin Effect on Cardiovascular Events–Thrombolysis in Myocardial Infarction 58; DAPA-HF: Dapagliflozin and Prevention of Adverse Outcomes in Heart Failure; PROactive: PROspective PioglitAzone Clinical Trial in MacroVascular Events; IRIS: Insulin Resistance Intervention After Stroke; HFrEF: heart failure with reduced ejection fraction; MI: myocardial infarction; TIA: transient ischemic attack

Category	Trial	Population & Design	Intervention	Key Outcomes	Limitations
Therapeutic convergence	EMPA-REG OUTCOME (Zinman et al., 2015) [116]	T2DM + established CVD; RCT	Empagliflozin	↓ CV death 38%; ↓ HF hospitalization 35%	Secondary infection risk
	CANVAS Program (Neal et al., 2017) [117]	T2DM + high CV risk; RCT	Canagliflozin	↓ MACE 14%; ↓ HF hospitalization	↑ Amputation risk signal
	DECLARE–TIMI 58 (Wiviott et al., 2019) [118]	T2DM with/at CV risk; RCT	Dapagliflozin	↓ HF hospitalization/CV death composite	Neutral on MACE
	DAPA-HF (McMurray et al., 2019) [119]	HFrEF ± diabetes; RCT	Dapagliflozin	↓ worsening HF/CV death 26%	HF population only
	PROactive (Dormandy et al., 2005) [120]	T2DM + macrovascular disease; RCT	Pioglitazone	↓ death/MI/stroke composite	Weight gain, edema
	IRIS (Kernan et al., 2016) [121]	Insulin resistance + stroke/TIA; RCT	Pioglitazone	↓ stroke or MI 24%	Fractures, fluid retention

Taken together, these converging advances in experimental modeling, biomarker development, genomic insights, and integrated care delivery outline a unified framework that links discovery science with clinical practice, emphasizing shared pathogenic pathways, stratified treatment guided by inflammatory phenotypes, and multidisciplinary care models as the foundation for reducing the dual burden of diabetes and CVD.

## Conclusions

This review establishes metabolic inflammation as the central mechanistic link between cardiovascular and endocrine disorders, emphasizing the interconnected roles of adipose tissue dysfunction, gut microbiota alterations, inflammasome activation, and mitochondrial oxidative stress in driving systemic disease. We highlighted the diagnostic and prognostic potential of inflammatory biomarkers, the therapeutic convergence of pharmacological and lifestyle interventions, and the promise of multi-omics and bioengineered models in advancing translational research. However, limitations such as heterogeneous evidence, lack of standardized biomarker thresholds, and insufficient population diversity constrain generalizability. Addressing these gaps through integrated clinical frameworks, stratified anti-inflammatory therapies, and patient-centered outcomes will be pivotal in establishing precision medicine strategies to mitigate the dual burden of cardiovascular and endocrine diseases.
